# Patient Impact of Surgery Abroad: A Retrospective Qualitative Analysis

**DOI:** 10.7759/cureus.92497

**Published:** 2025-09-16

**Authors:** David J Solomon, Victoria R Marks, Mia Luke, Michael H Eames, Kevin J Donnelly

**Affiliations:** 1 Trauma and Orthopaedics, Ulster Hospital, Belfast, GBR; 2 Trauma and Orthopaedics, Queen’s University Belfast, Belfast, GBR

**Keywords:** abroad, complications, fracture, orthopaedic emergency, trauma

## Abstract

Background

With increasing international travel, more Northern Ireland (NI) residents are sustaining injuries abroad, sometimes requiring emergency orthopaedic surgery. Upon repatriation, the National Health Service (NHS) must address complications and ensure continuity of care. This study evaluates the experiences and outcomes of NI residents who underwent emergency orthopaedic surgery abroad.

Methods

A retrospective qualitative descriptive study was conducted at Ulster Hospital, Belfast, Northern Ireland, between August 2024 and April 2025. Patients who sustained orthopaedic injuries abroad and received emergency surgery overseas were identified via Virtual Fracture Clinic (VFC) records. Data were collected through structured online surveys and analysed descriptively.

Results

Twelve patients were included (median age 62.5 years; nine female, three male). Procedures included open reduction and internal fixation (ORIF) (n = 8), intramedullary (IM) nail (n = 3), and total shoulder arthroplasty (TSA) (n = 1). Postoperative complications occurred in seven patients (58%), including infection, non-union, hardware failure, and the need for revision surgery. Communication barriers were common: two patients did not sign consent forms, and half of those in non-English-speaking countries lacked interpreter support. Four patients felt pressured into surgery abroad, and six (50%) would have preferred repatriation to NI for treatment.

Discussion

Overseas surgery was associated with a high complication rate, substantially exceeding that reported for similar procedures in the UK (10%-20%). Contributing factors included inadequate consent, poor communication, limited postoperative documentation, and lack of structured discharge planning. These findings indicate that emergency surgery abroad frequently compromises patient safety and increases the NHS resource burden.

Conclusion

Emergency orthopaedic surgery abroad carries significant risks, with complications nearly three times higher than UK benchmarks. Improvements in international communication, interpreter availability, and repatriation protocols are urgently needed. NHS policy should anticipate the rising demand for coordinated, cross-border care.

## Introduction

International travel among Northern Ireland (NI) residents has risen sharply, with 1.41 million trips recorded in 2017 - an increase of 71% since 2013. With this rise comes a parallel increase in injuries sustained abroad, many of which require urgent surgical intervention. Fractures, dislocations, and soft tissue injuries are common, often necessitating emergency orthopaedic surgery prior to repatriation [1,2].

Unplanned overseas surgery raises complex issues around continuity of care, informed consent, and communication. While literature on medical tourism exists, research into emergency surgery abroad is sparse. This study investigates the patient experience and outcomes of NI residents undergoing emergency orthopaedic surgery abroad, with a focus on postoperative complications and the subsequent burden on the National Health Service (NHS) [3,4].

## Materials and methods

Study design and participants

A retrospective qualitative descriptive study was conducted at Ulster Hospital, Belfast, Northern Ireland, analysing the experiences of NI patients undergoing emergency orthopaedic surgery abroad. This design was selected as it allowed for exploration of patient experiences and identification of themes relating to safety, communication, and continuity of care. All participants provided informed consent via telephone prior to survey participation. All patients were identified via the Ulster Hospital Virtual Fracture Clinic (VFC) between August 2024 and April 2025.

Inclusion and exclusion criteria

(I) All NI residents who sustained an orthopaedic injury outside a UK NHS hospital; (II) patients who underwent emergency surgery overseas; and (III) subsequently presented to Ulster Hospital on return.

(I) Patients with elective procedures abroad; (II) non-operative fracture management overseas; (III) refusal to participate; and (IV) patients presenting to other Northern Irish hospitals during this time period. 

Survey development and content

A 14-question survey was designed by the senior authors of this study and reviewed by consultant orthopaedic surgeons within Ulster Hospital to ensure face validity and content relevance. The survey comprised a mix of fixed-response questions and free-text options. Domains included patient demographics; location of injury and surgery; consent processes; interpreter access; quality of communication and risk explanation; perceived pressure to undergo surgery; financial implications; timing of surgery; repatriation preferences; and postoperative experiences. Specifically, patients were asked to rate the clarity of injury explanation, risk disclosure, and discussion of alternatives on a 1-10 scale. Two questions permitted free-text comments to capture additional insights (Table 3, see Appendix).

Survey distribution

Patients were first contacted via telephone to confirm eligibility and obtain verbal consent. A survey link (Google Forms; Google, Inc., Mountain View, CA, USA) was then sent by text message. Reminder text messages were issued one week later to non-responders, followed by a further telephone call at two weeks to maximise participation. Of the 13 eligible patients contacted, 12 completed the survey (response rate 92%).

Data collection and outcomes

Survey responses were collected electronically and cross-checked against hospital records to verify surgical procedures, complications, and follow-up. The primary outcome was the rate of postoperative complications, categorised as major (requiring return to theatre or multidisciplinary intervention) or minor (managed conservatively). Secondary outcomes included patient-reported experiences of communication, consent, interpreter use, discharge documentation, financial costs, and preferences for repatriation.

Analysis

Quantitative data were summarised using descriptive statistics. Qualitative responses were analysed thematically, grouping patient experiences into categories: communication barriers, autonomy/consent, continuity of care, and repatriation/financial challenges [5-7].

## Results

From the patients responding to the survey, 12 patients (median age 62.5 years, 75% female) underwent overseas emergency orthopaedic surgery. Open reduction and internal fixation (ORIF) was the most common (n = 8), followed by intramedullary (IM) nail (n = 3) and total shoulder arthroplasty (TSA) (n = 1), the demographics of which are outlined in Table 1.

**Table 1:** Patient Demographics Table showing patient demographics of patients operated on abroad between August 2024 and April 2025. ORIF, Open Reduction and Internal Fixation; IM Nail, Intramedullary Nail; TSA, Total Shoulder Arthroplasty

Characteristic	Value	N	%
Total Patients	12	12	100
Median Age (years)	62.5 (33-75)	-	-
Gender	Female	9	75
	Male	3	25
Procedure	ORIF	8	66.7
	IM Nail	3	25
	TSA	1	8.3
Injury Site	Lower Limb	8	66.7
	Upper Limb	4	33.3

Complications were reported in seven patients (58%), as seen in Table 2. Major complications included deep surgical site infection, hardware failure with non-union, protruding metalwork requiring revision, and the need for surgical debridement. One case required return to theatre for combined orthopaedic and plastic surgery intervention for flap coverage. Minor complications included persistent pain, stiffness, wound hypersensitivity, and superficial infection. Overall, four patients required return to theatre following their initial overseas procedure.

**Table 2:** Postoperative Complications Table outlining complications following surgery abroad.

Category	N	%
Major complications	4	33.3
Minor complications	3	25.0
No complications	5	41.7

Furthermore, two patients did not sign a consent form prior to surgery, and one was unsure of the procedure performed. Half of those treated in non-English-speaking countries lacked interpreter support. Four patients reported feeling pressured into surgery abroad, and six would have preferred repatriation to NI for treatment. Documentation at discharge was inconsistent: only 9 out of 12 patients received operative or postoperative notes.

## Discussion

This study demonstrates a complication rate of 58% following emergency orthopaedic surgery abroad - markedly higher than UK benchmarks, where infection rates after fracture fixation are usually 5%-8%, and non-union is reported in 2%-10% of cases [8,9]. The frequency of return to theatre, infection, non-union, and hardware failure observed in our cohort suggests deficiencies in perioperative standards, consent processes, and postoperative care delivered outside the UK. These findings align with wider concerns that surgery abroad may compromise patient safety and subsequently increase the burden on the NHS [10].

Communication and informed consent emerged as significant issues, as outlined by the General Medical Council (GMC) [11,12]. Half of the patients treated in non-English-speaking countries lacked interpreter support, and in some cases, family members acted as informal translators. Two patients did not sign consent forms at all, and others described inadequate explanation of risks or alternatives. Although all patients received some description of their injury, their understanding of complications and treatment options was often superficial. Several reported feeling pressured into surgery, with decision-making shaped more by urgency than by genuine informed choice. Such patterns highlight how linguistic and systemic barriers abroad may undermine autonomy and informed consent, exposing patients to procedures without a clear grasp of potential risks or outcomes.

Continuity of care and discharge planning were also weak. Half of the patients reported little or no postoperative plan, with limited documentation provided on discharge. Those who required follow-up in NI often returned with scant operative notes, impeding the smooth transition of care. The absence of structured communication between overseas centres and the NHS created delays in physiotherapy, confusion over metalwork removal, and difficulty in planning rehabilitation. These findings mirror wider literature, showing that poor discharge coordination in cross-border settings increases the risk of delayed recovery and complications [13,14].

Repatriation preferences further underline the limitations of overseas care. Half of the cohort stated they would have preferred to return home for surgery, including all four patients who reported feeling pressured into procedures abroad. Barriers to repatriation included financial cost, lack of insurance clarity, and uncertainty over eligibility for transfer once treatment had started abroad. Several patients incurred out-of-pocket expenses, while others struggled with reimbursement. These gaps between entitlement and access meant that patients were often left with no practical option but to undergo surgery abroad, even when they would have preferred NHS care.

The consequences for the NHS were substantial. Four patients required revision surgery at Ulster Hospital, including one requiring joint orthoplastic input, while others presented with issues such as screw prominence, hypersensitivity, delayed healing, malunion, or intra-articular screw placement requiring removal.

In elective cosmetic tourism, average NHS costs for revision procedures have been estimated at £9,000-£12,500 per case, and while this study focuses on emergency surgery, the parallels are clear. Complex revision surgery, infection management, and prolonged rehabilitation add significant strain to an already pressured system [15].

The broader phenomenon of medical tourism highlights the same themes. Studies have shown that complications from surgery abroad frequently present to NHS services, often with incomplete documentation and at considerable cost. Infections, delayed unions, and implant failures all require high-resource interventions, with costs several times greater than those associated with uncomplicated healing [16,17]. Our findings reflect this wider pattern and underscore the importance of anticipating the clinical and economic burden associated with cross-border emergency surgery.

Overall, these results reinforce the need for greater awareness of the risks associated with undergoing emergency surgery abroad. They also highlight system-level deficiencies that extend beyond individual cases: inadequate interpreter provision, superficial consent processes, absent discharge planning, financial obstacles to repatriation, and lack of coordination between health systems [18]. The British Orthopaedic Association’s 2025 theme of “Preventing Harm” is particularly relevant here, as these harms are predictable, preventable, and increasingly common in the context of rising international travel [19].

There needs to be further guidance for patients on the risks of overseas surgery. One possible method of improving care could be via organised ambulatory care on return to NI. Due to advances in technology, advice can now be given by NHS experts via remote consultations. Structured repatriation pathways and public health messaging could improve patient safety. Anticipating these rising overseas injuries is essential to reducing avoidable harm. Furthermore, policy implications include the need for international collaboration on documentation, discharge communication, and interpreter provision.

Limitations

This study has several limitations. The sample size was small (n = 12), limiting generalisability. The retrospective design relied on survey responses, introducing potential recall bias. Inconsistencies in overseas documentation may have led to incomplete information. Furthermore, this was a single-centre study in NI, and the results may not be representative of all NHS settings. Despite these limitations, the findings highlight important safety and policy concerns.

Future work should focus on larger, multi-centre studies and include economic modelling to define the cost burden more accurately. International collaboration on discharge documentation, repatriation protocols, and patient-facing travel health guidance will be critical to improve continuity of care. Without such measures, patients will remain exposed to unnecessary risks abroad, and the NHS will continue to bear the consequences of complications originating outside its system.

## Conclusions

Emergency orthopaedic surgery abroad carried a complication rate nearly three times higher than UK benchmarks, with adverse outcomes in nearly 60% of patients. Inadequate communication, limited consent, and poor continuity of care contributed significantly to these results. The consequences extend beyond patients, creating a notable burden on the NHS, both in terms of cost and clinical resources.

Ultimately, opting for surgery abroad - particularly under emergency circumstances - appears to be associated with substantially worse outcomes than treatment within the UK. Addressing this issue will require coordinated action at both national and international policy levels.

## Appendices

**Table 3:** Patient Survey Questionnaire Used in This Study The following survey was administered to all eligible patients; it included 14 items and was sent via a text message link to Google Forms.

No.	Survey Question	Response Format
1	What is your date of birth?	Free text (DD/MM/YYYY)
2	Where did your surgery take place? (Country/City)	Free text
3	Were you provided with an interpreter?	Yes/No
4	Were you asked to sign a consent form?	Yes/No
5	On a scale of 1-10, how clearly was your injury explained to you?	1-10 scale
6	On a scale of 1-10, how clearly were the risks of surgery explained to you?	1-10 scale
7	On a scale of 1-10, how clearly were alternative treatment options explained to you?	1-10 scale
8	Did you feel pressured into having the operation?	Yes/No
9	How soon after your injury did the surgery take place?	Free text (number of days/hours)
10	Did you have to pay for your surgery abroad?	Yes/No (with free text option for amount/insurance details)
11	Would you have preferred to return home (NI) for surgery if given the choice?	Yes/No
12	What complications, if any, did you experience after surgery?	Free text
13	Were you provided with any discharge documentation or follow-up plan?	Yes/No/Free text
14	Any additional comments about your experience?	Free text
